# Analysis of Genomic Regions of *Trichoderma harzianum* IOC-3844 Related to Biomass Degradation

**DOI:** 10.1371/journal.pone.0122122

**Published:** 2015-04-02

**Authors:** Aline Crucello, Danilo Augusto Sforça, Maria Augusta Crivelente Horta, Clelton Aparecido dos Santos, Américo José Carvalho Viana, Lilian Luzia Beloti, Marcelo Augusto Szymanski de Toledo, Michel Vincentz, Reginaldo Massanobu Kuroshu, Anete Pereira de Souza

**Affiliations:** 1 Centro de Biologia Molecular e Engenharia Genética (CBMEG), Universidade Estadual de Campinas, Campinas, São Paulo, Brazil; 2 Instituto de Biologia (IB), Universidade Estadual de Campinas, Departamento de Biologia Vegetal, Campinas, São Paulo, Brazil; 3 Instituto de Ciência e Tecnologia, Universidade Federal de São Paulo, São José dos Campos, São Paulo, Brazil; Clemson University, UNITED STATES

## Abstract

*Trichoderma harzianum* IOC-3844 secretes high levels of cellulolytic-active enzymes and is therefore a promising strain for use in biotechnological applications in second-generation bioethanol production. However, the *T*. *harzianum* biomass degradation mechanism has not been well explored at the genetic level. The present work investigates six genomic regions (~150 kbp each) in this fungus that are enriched with genes related to biomass conversion. A BAC library consisting of 5,760 clones was constructed, with an average insert length of 90 kbp. The assembled BAC sequences revealed 232 predicted genes, 31.5% of which were related to catabolic pathways, including those involved in biomass degradation. An expression profile analysis based on RNA-Seq data demonstrated that putative regulatory elements, such as membrane transport proteins and transcription factors, are located in the same genomic regions as genes related to carbohydrate metabolism and exhibit similar expression profiles. Thus, we demonstrate a rapid and efficient tool that focuses on specific genomic regions by combining a BAC library with transcriptomic data. This is the first BAC-based structural genomic study of the cellulolytic fungus *T*. *harzianum*, and its findings provide new perspectives regarding the use of this species in biomass degradation processes.

## Introduction

Second-generation biofuels are among the key technologies being used in the decarbonization of the transportation sector [1], and second-generation bioethanol has emerged as a profitable alternative to fossil fuels, as it can be used as either a substitute fuel (hydrous alcohol) or as an additive to gasoline (anhydrous alcohol) [2]. Brazil is the largest producer of sugarcane worldwide, producing 652 million tons in 2013 [3]. One-third of plant biomass consists of bagasse, which is a potential source of lignocellulosic material for second-generation bioethanol. In the first-generation bioethanol-production chain, ~92% of the bagasse is combusted for heat generation [4]. It is estimated that using the remaining 8% of the bagasse for the production of second-generation bioethanol would decrease land-use needs by 29% in Brazil [5].

Despite its advantages, the cost to produce ethanol from cellulose makes it impractical for large-scale production. There is therefore a need to better understand biomass hydrolysis, including the identification of novel genes and regulatory factors from cellulase-producing organisms.

The enzymatic hydrolysis of biomass is achieved via the synergistic action of several enzymes that are often secreted by microorganisms such as bacteria and fungi. Plant cell walls are composed of cellulose, hemicelluloses, pectins, lignin, and other molecules [6]. The following three types of cellulases are required for cellulose hydrolysis: *a)* endoglucanases, which randomly cleave the internal bonds of polysaccharides possessing β-1,4-glucan backbones; *b)* cellobiohydrolases, which are responsible for cleaving the terminal chains produced by endoglucanases and release molecules containing 2 or 4 glucose units; and *c)* β-glucosidases, which hydrolyze β-glycosidic bonds from cellobiose and other oligosaccharides to form glucose. However, several relevant enzymes and regulatory elements are involved in lignocellulose hydrolysis, some of which have not been identified.

*Trichoderma harzianum* (teleomorph *Hypocrea lixii*) is an ascomycete fungus that is ubiquitous in the environment. This species is used in the biological control of a variety of plant-pathogenic fungi and shows a plant growth-promoting ability [7, 8]. Enzymes from *T*. *harzianum* are employed in the food (9), textile and paper industries [9, 10]. Additionally, it has been known since the 1980s that *T*. *harzianum* secretes a number of biomass-degrading enzymes, including cellulases and hemicellulases [11]. However, a limited number of studies addressing the use of the enzymes of this species for lignocellulose hydrolysis have been conducted compared with other organisms such as *Trichoderma reesei*.

Castro et al. (2010) [12] cultivated *T*. *harzianum* IOC-3844, a publically available Brazilian strain, in pretreated sugarcane bagasse and verified high levels of endoglucanase activity, in addition to significant levels of β-glucosidase and *FPase* (Filter Paper Activity), demonstrating that this strain exhibits appropriate characteristics for application in cellulose hydrolysis. Subsequent studies characterized important cellulases that are secreted by this strain, e.g., cellobiohydrolase 1 (Cbh1) and endoglucanase 3 (Egl3) [13–15]. Additionally, Horta et al. (2014) [16] outlined the transcriptomic profile of the IOC-3844 strain and identified the entire set of expressed genes in a cellulosic substrate. Although efforts have been made to identify the transcriptomic features of this fungus, the literature lacks information from genomic-level analyses and on genes related to biomass degradation. Therefore, this study aims to investigate the genomic context of the *T*. *harzianum* biomass degradation genes by screening for novel candidate regulatory genes using transcriptome data. This work also extends the analysis to a genomic level via bacterial artificial chromosome (BAC) library construction.

The BAC vector is based on the F-factor, a single-copy plasmid found in *E*. *coli* that is related to fertility [17]. This system is capable of cloning large DNA inserts and is employed for gap filling at the genome level as well as for physical mapping and the preservation of genetic resources [18]. Considering possible insert sizes of approximately 100 kbp and the speed of this technique (less than two weeks to obtain a whole-genome library), we chose to use this system to analyze genes situated in proximity to those related to biomass degradation in *T*. *harzianum*.

Seven CAZy (carbohydrate-active enzymes, www.cazy.org) genes, which are known for their substantial role in biomass hydrolysis, were selected for the analysis. Employing a rapid selection platform using φ-29 polymerase, BAC clones containing the selected genes were identified and sequenced with 454 technology. Following assembly and annotation, the transcriptomic data were used to delineate the expression profile of all of the genes identified in each BAC. Our results revealed genomic regions that were rich in CAZy genes, with candidate regulatory genes presenting expression profiles similar to those of CAZy genes. In combination with transcriptome data, we used a BAC library, which permits the selection of large genomic fragments (~90 kbp in the present work) with high clonal stability, to demonstrate the application of a rapid tool for the screening of genes and regulatory factors. These techniques enabled a comprehensive analysis of the target genomic regions of *T*. *harzianum* IOC-3844.

## Materials and Methods

### Strains, media, and chemicals

*T*. *harzianum* IOC-3844 was provided by Dr. Nei Pereira, Jr. (Federal University of Rio de Janeiro—Rio de Janeiro, Brazil). The variability of the fungal ribosomal ITS1 and ITS2 sequences was used for species confirmation via comparison with the sequences present in the standard strains of *T*. *harzianum* (available at the Oswaldo Cruz Institute, Rio de Janeiro, RJ, Brazil).

The strain was maintained on potato dextrose agar (PDA; 200 g potato, 20 g dextrose, 20 g agar, 1,000 mL distilled water, pH 6.5) at 28°C and stored in PDA slants at 4°C. Prior to BAC library construction, the fungus was grown on PDA plates at 28°C for 5 days for conidia production. The fungal conidia (1 mL of a suspension containing 1 × 10^6^ conidia/mL in sterile distilled water containing 1% Tween 80 v/v) were grown in 100 mL of potato dextrose broth (PDB) for 16 h at 28°C at 200 rpm before preparing the protoplasts for the isolation of high molecular weight (HMW) DNA. All of the chemicals and media components were of analytical grade.

### High molecular weight (HMW) DNA preparation

Protoplasts were generated following previously described protocols, with some modifications [19, 20]. After 16 hours of conidial incubation in PDB, young germlings were harvested via filtration through sterile Miracloth (Merck KGaA, Darmstadt, Germany) and washed once with sterile distilled water. This step was followed by two washes with an osmotic stabilizer (0.6 M KCl, pH 5.6). The culture was then re-suspended in Driselase (Sigma-Aldrich, USA) solution (5 mg/mL in 0.6 M KCl) and incubated at 28°C at 180 rpm for 3 hours. After incubation, the culture was filtered through Miracloth into a centrifuge tube to remove mycelial fragments. The culture was then washed twice with 20 mL of osmotic stabilizer to remove enzyme remnants, followed by centrifugation at 500 *g* for 5 min at 4°C. The final protoplast pellet was re-suspended in 2 mL of 0.6 M KCl (pH 6.5) at a concentration of ~1 × 10^8^ spores/mL. The protoplasts were embedded in 0.75% LMP agarose (Lonza) plugs. Protoplast lysis to release HMW DNA was performed according to the methods described by Peterson et al. (2000) [21]. The plugs were stored in 0.05 M EDTA, pH 8.0, at 4°C for up to four weeks.

### Enzyme kinetics and size selection of HMW DNA

To ensure the isolation of DNA of the expected size, two partial restriction digests were performed to determine the conditions yielding an appropriate percentage of fragments between 100 and 350 kb in size. Size selection was performed according to previously described protocols using the *Hind*III enzyme (New England Biolabs, UK) [19, 21]. The optimal conditions were determined by digesting the chopped plugs at several enzyme concentrations (0.2–50 U). The macerated plug pieces were placed in 1.5-mL Eppendorf tubes containing the appropriate buffer and the *Hind*III enzyme and incubated on ice for 1 hour. Digestion was performed at 37°C for 10 min, and the reaction was stopped by adding 30 μL of 0.5 M EDTA (pH 8.0).

Partially digested HMW DNA was separated via PFGE (pulsed-field gel electrophoresis) in 1% TBE agarose (CHEF-DR II drive module, Bio-Rad) using the following parameters: 120° angle, 12°C, 6.0 V/cm, initial switch time = 1.0 sec, final switch time = 40.0 sec, ramping = linear, and running time = 18 hours. Once the optimal *Hind*III concentration was determined, mass digestion was performed using 13 plugs. The digested HMW DNA was separated into two selected sizes via PFGE, as described by Peterson et al. (2000) [21].

The DNA inserts embedded in agarose gel pieces were maintained in 50-mL polypropylene centrifuge tubes containing 1× TAE at 4°C before electroelution.

### Construction of the BAC library

HMW insert DNA was removed from the agarose gel slices via electroelution, as previously described [21]. The DNA concentration was determined in agarose gel via comparison with various concentrations of an uncut lambda DNA marker. Ligation was performed using the vector pIndigoBAC-5 (*Hind*III-Cloning Ready, Epicentre Biotechnologies) and the insert DNA at a ratio of 3:1 insert/vector in a 100-μL reaction volume at 16°C for 10 hours, followed by enzyme inactivation at 65°C for 20 min. The ligation mixture was desalted for 1 hour in 1% agarose and 100 mM glucose on ice. The desalted mixture (~ 65 μL) was divided into 5 equal parts and transformed into electrocompetent *E*. *coli* DH10B phage-resistant cells (Invitrogen) using a MicroPulser Electroporator (Bio-Rad) with programmed function Ec3 (cuvette size: 0.2 cm; 3.00 kV; 1 pulse). The transformed cells were immediately resuspended in 2 mL of pre-heated (37°C) SOC medium and incubated at 37°C for 1 hour, with shaking at 250 rpm. The obtained transformants were grown at 37°C overnight in LB (20 g tryptone, 10 g yeast extract, 10 g NaCl, distilled water up to 1000 mL, pH 7.2) supplemented with 12.5 mg/mL chloramphenicol, IPTG, and X-Gal.

The recombinant white colonies were selected using Qpix2 (Genetix) and placed in 384-well plates containing LB with 6% glycerol and 12.5 mg/mL chloramphenicol. The plates were incubated overnight at 37°C at 350 rpm and stored at -80°C.

### Insert size analysis

To determine the insert size present in the *T*. *harzianum* IOC-3844 BAC library, BAC DNA was isolated from 90 randomly selected clones cultured in 1.2 mL of LB medium with 12.5 mg/mL chloramphenicol overnight using the NucleoSpin 96 Flash kit (Macherey-Nagel 740618.24), which is suitable for the high-throughput purification of large constructs. BAC DNA was digested with *Not*I (New England Biolabs, UK) at 37°C for 6 hours and analyzed via PFGE under the following conditions: 6.0 V/cm; initial switch time = 5.0 sec; final switch time = 15.0 sec; included angle = 120°; 12°C; ramping = linear; running time = 16 hours.

### BAC end sequencing

Randomly selected BAC clones (n = 84) were inoculated in 2 LB medium (20 g tryptone, 10 g yeast extract, 10 g NaCl, distilled water to 1000 mL, pH 7.2) with 12.5 mg/mL chloramphenicol. BAC DNA was isolated using NucleoSpin 96 Flash (Macherey-Nagel 740618.24) following the manufacturer’s instructions. The DNA template was subjected to a cycle sequencing reaction with the M13/T7 BAC-END primers (M13: 5’-AACAGCTATGACCATG-3’; T7: 5’-TAATACGACTCACTATAGG-3’) using the Big-Dye v3.1 (Life Technologies) terminator. The reactions were performed in a 15-μL volume (5 μL of 300 ng/μL BAC DNA, 0.5 μL of Big-Dye, 2.0 μL of 5× sequencing buffer, 0.5 μL of primers, and 7.0 μL of deionized water). PCR was conducted in a PTC-200 Thermal Cycler (Bio-Rad) using the following program: 95°C for 1 min, followed by 90 cycles of 95°C for 20 sec, 50°C for 20 sec, and 60°C for 4 min. The reactions were purified via EDTA/ethanol precipitation and analyzed using an ABI PRISM 3500 sequencer. The BAC end sequences were trimmed and subjected to BLAST searches against the *T*. *harzianum* CBS 226.95 v1.0 scaffolds from the JGI (Joint Genome Institute) to estimate the overall distribution of random clones within the genome. Only unique hits were considered.

### Screening of cellulase-containing clones—Rapid selection platform

For screening, a rapid selection platform was used based on multiple displacement amplification (MDA) [22]. Briefly, 2-μL aliquots from each of the 384 clones (corresponding to 1 plate) were pooled (plate pool) in 20 mL of LB medium (6% glycerol and 12.5 mg/mL chloramphenicol) and incubated for 20 to 22 hours at 37°C and 40 rpm. The procedure was repeated for all 15 plates, and a 1-mL aliquot from each plate pool was stored at -80°C. The plate pools were amplified using the Illustra GenomiPhi HY DNA Amplification Kit (GE Healthcare Life Sciences, UK) following the manufacturer’s instructions.

As previous studies have revealed high levels of endoglucanase and xylanase production, significant β-glucosidase activity, and potential biotechnological applications for cellobiohydrolase I from *T*. *harzianum* IOC-2844 [12, 14, 15], the following 7 genes related to biomass degradation were selected for screening: 3 endoglucanases (Egl1, Egl2, Egl3), 1 cellobiohydrolase (Cbh1), 1 swollenin (Swo), 1 β-glucosidase (Bgl2), and 1 xylanase (Xyn2). Primers were designed (S1 Table) for each gene to select BAC clones for 454 sequencing. PCR was performed for each of the 15 amplified plate pools for the 7 genes, and positive plates were subjected to individual clone screening via PCR using the CFX384 Touch Real-Time PCR Detection System (Bio-Rad).

### Sequencing and assembly of the selected BAC clones

Briefly, 454 GS FLX sequencing was performed by the Center for Advanced Technologies in Genomics (CATG) at the University of São Paulo, São Paulo, SP, Brazil. The generated reads were *de novo* assembled using the Phred, Phrap, and Consed programs, with a quality value of 20 being produced by the Phred base caller. Cross-matching was used to mask the cloning vector and other possible *E*. *coli* sequences. The assembled BACs from the same screened target gene were aligned to obtain a larger BAC.

### Gene prediction and functional annotations

The online tools FGENESH [23] and Augustus [24] were used for initial gene prediction analysis, followed by manual annotation using the Artemis Genome Browser [25]. The predicted amino acid sequences were subjected to BLAST searches against the UniProtKB/Swiss-Prot database and were manually corrected when necessary. Automated annotations were performed with Blast2GO [26] using BLASTx with an E-value threshold of 10^–6^. All of the predicted gene models were functionally annotated based on homology to annotated genes from the NCBI non-redundant dataset and classified according to the Gene Ontology and the InterPro signatures databases [27, 28].

### Expression pattern

The predicted BAC genes were analyzed with respect to their expression levels using RNA-Seq data obtained from previous work [16] in which the transcripts were obtained through fungal growth on three carbon sources: lactose (LAC), cellulose (CEL), and delignified sugarcane bagasse (DSB). The reads from the RNA-Seq library were mapped against BAC genes using CLC Genomics Workbench (CLC bio—v4.0; Finlandsgade, Dk) with the following parameters: mapping settings (minimum length fraction = 0.7, minimum similarity fraction = 0.8, and maximum number of hits for a read = 1) and paired settings (minimum distance = 180 and maximum distance = 1000, including the broken pairs counting scheme).

The expression values were expressed in RPKM (reads per kilobase of exon model per million mapped reads). The predicted BAC genes were clustered with a K-means algorithm using CLC bio. The clustering was performed applying Euclidian distance as the distance metric in 10 partitions (*k* = 10) for the predicted genes for each BAC according to the cluster features of the log_2_-transformed expression values. K-means clustering for CAZy genes was performed with the same features, except for the number of partitions (*k*_=_ 6). A hierarchical clustering analysis was conducted with CLC-bio using the single linkage method and Euclidian distance.

### Real-Time quantitative qRT-PCR analysis

The expression levels of the BAC genes that were found in the RNA-Seq library were validated via quantitative real-time PCR (qRT-PCR). DNaseI-treated total RNA was employed to synthesize first-strand cDNA using the Promega ImProm-II Reverse Transcription System and oligo(dT)_18_ according to the manufacturer’s instructions.

Five CAZy genes (Egl1, Egl2, Egl3, Swo, and Xyn2) were selected for the qRT-PCR analysis. A squalene epoxidase gene (*erg1*) from *T*. *harzianum*, which is involved in ergosterol biosynthesis, was used as the reference gene and was previously established as the best endogenous control gene for the *T*. *harzianum* IOC-3844 strain [16]. The primers for all of the genes are provided in S2 Table. The reactions were performed in duplicate in a 384-well plate containing 3.12 μL of SYBR Green Master Mix (Invitrogen, Carlsbad, CA), 1.0 μL of the forward and reverse primers, 0.5 μL of cDNA, and 1.6 μL of deionized water, totaling 6.25 μL.

qRT-PCR was conducted in the CFX384 Touch Real-Time PCR Detection System (Bio-Rad). The real-time PCR program was as follows: initial denaturation at 95°C for 10 min, followed by 40 cycles of 15 sec at 95°C and 60 sec at 60°C. All of the PCR amplifications were performed in duplicate, and DNase-treated RNA was used. In addition, a control without reverse transcriptase was included. The specificity of the amplification was verified using melting curves. Gene expression was calculated via the Delta-Delta cycle threshold method [29]. All statistical comparisons were performed using Student’s t-test (P<0.05).

## Results and Discussion

### Construction and characterization of a *T*. *harzianum* BAC library

We constructed and characterized a BAC library from the hyper-cellulolytic fungus *T*. *harzianum* IOC-3844. The library consisted of 5760 clones that provided 12-fold coverage of the fungal genome.

**Fig 1:**
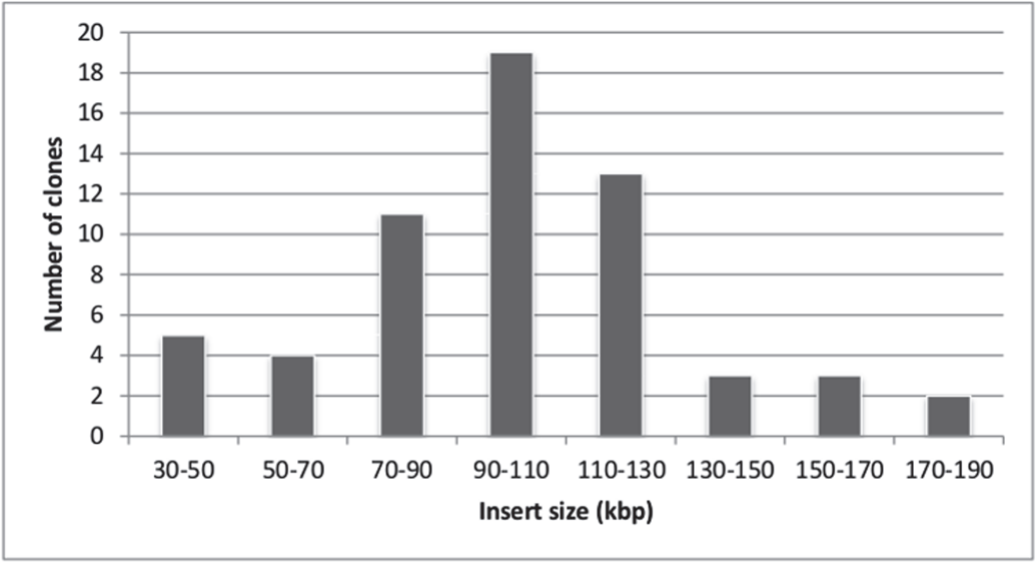
Insert size distribution in the *T*. ***harzianum* IOC-3844 BAC library**. Sixty randomly selected BAC clones were digested by *Not*I. The insert size ranged from 35 to 180 kbp, with an average size of 90 kbp.

The average insert size in the library was determined via *Not*I digestion of 84 randomly selected clones. All of the analyzed clones contained inserts. The estimated insert size ranged from 35 to 180 kbp, with an average size of 90 kbp (Fig. 1). With a total of 5760 clones (15 × 384-well plates) and a mean size of 90 kbp, the total library contained approximately 518,400 Mb of *T*. *harzianum* genomic DNA. According to the genome assembly of the CBS 226.95 strain v1.0 release from the JGI, the haploid size of the *T*. *harzianum* genome is 40.98 Mb. Assuming that the genome size of the CBS 226.95 strain is similar to that of IOC-3844, our BAC library is estimated to contain 12 genome equivalents. We confirmed the library coverage through the PCR amplification of two single-copy genes (*egl1* and *egl3*), which amplified 10 clones each, thus suggesting that the library covers the fungal genome by ~ 10 to 12 fold.

To test the representativeness of the library, BAC end sequencing was performed using 84 clones. BAC end sequences (BESs) ranging from 400 to 600 bp were subjected to BLAST searches against the *T*. *harzianum* CBS 226.95 genome (scaffolds) with an E-value cutoff of 1e-5. All of the 84 BESs were successfully mapped to *T*. *harzianum* CBS 226.95 sequences, which were allocated among 23 different scaffolds, ranging from 235 kb to 4.09 Mb in length, and covering ~ 83% of the fungal genome. As verified through the wide range of the mapped scaffolds, the constructed BAC library showed good representation.

### Rapid selection of clones

**Fig 2:**
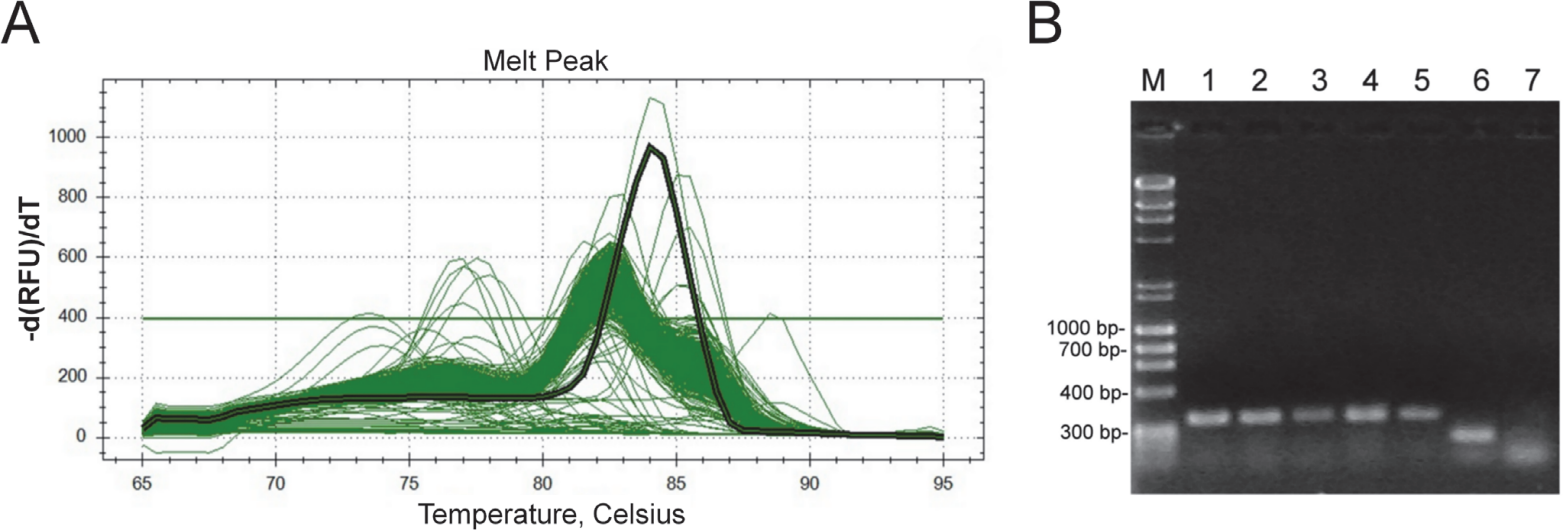
Screening of the Egl1-positive plate. Positive-pool plates were screened via PCR using the CFX384 Touch Real-Time PCR Detection System (Bio-Rad). The positive clones were identified based on the melt peak temperature that was previously established for each primer pair. Panel **A** shows a highlighted melt peak, indicating a positive clone for Egl1 (melt temperature = 84°C). The positive clones were confirmed through agarose gel visualization, as shown in panel **B.**

Amplified DNA samples from the 15 plates were screened using a specific set of primers for 7 genes related to biomass degradation (S1 Table). The primers were designed based on the *T*. *harzianum* IOC-3844 transcriptome [16]. For the Egl1 gene, 5 plates exhibited amplification. The positive plates for each gene were then screened individually, as shown in Fig. 2A. The positive products of PCR amplification of the selected clones were validated via agarose gel electrophoresis (Fig. 2B). A total of 38 clones were selected: 10 clones for *egl*1, 10 for *egl*3, 6 for *egl*2, 3 for *bgl*, 3 for *cbh*1, 3 for *swo*, and 3 for *xyn2*.

Prior to 454 sequencing, at least 3 clones from each gene were fingerprinted using both the *Hind*III and *Xho*I enzymes (data not shown) to ensure that the BAC fragments did not completely overlap. Fingerprinting is an important step after sequencing to enable the assembly of larger genomic regions of interest. This step increases the chance that the target genes are not in close proximity to the BAC ends.

### 454 sequencing and BAC insert assembly

Eighteen BAC clones were selected for sequencing. Pyrosequencing of the 18 BAC clones yielded 694,226 reads with a mean length of 354 bp, generating ~150× coverage of the sequenced DNA. The swollenin BAC aligned with Cbh1; thus, we obtained 6 different sequenced genomic regions. The assembly results are summarized in Table 1, and the corresponding GenBank accession numbers for each BAC sequence are: (KM555248), (KM555249), (KM555250), (KM555251), (KM555252), and (KM555253).

**Table 1:** Assembly results for the 454-sequenced clones.

**BAC (Assembled BAC size)**	**BAC code**	**Contig size (kbp)**
**Cbh1 (146 kbp)**	AFP10C24	87
	AFP10F23	96
	AFP13C12	71
**Bgl2 (161 kbp)**	BGP10N17	84
	BGP1J16	93
	BGP3E20	80
**Egl1 (165 kbp)**	E1P11O8	110
	E1P1I5	116
	E1P5B12	95
**Egl2 (153 kbp)**	E2P1G16	84
	E2P3O6	31
	E2P8I9	99
**Egl3 (147 kbp)**	E3P1M21	101
	E3P2I23	87
	E3P3J17	106
**Swo (146 kbp)** ^**a**^	SWP5C20	95
	SWP5L15	123
**Xyn2 (113 kbp)**	XIP1G10	113

^a^ The swollenin BAC aligned with the cellobiohydrolase I BAC; thus, the assembled BAC of ***swo*** and ***cbh1*** is the same for both screened genes.

### Annotation of BAC genes

**Fig 3:**
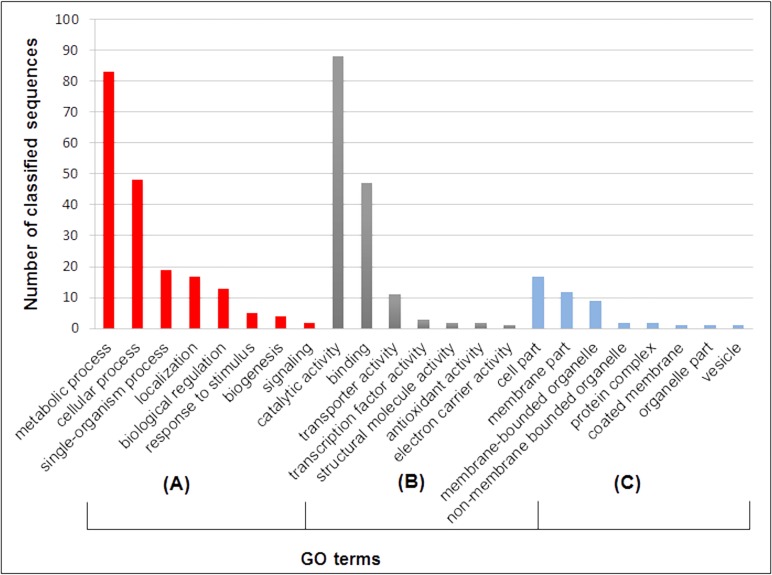
GO term distribution among the annotated BAC sequences. **(A)** According to biological processes; (**B)** According to molecular functions; and **(C)** According to cellular components.

Within the 6 assembled *T*. *harzianum* IOC-3844 BACs, 232 genes were predicted, with an average of 38.6 genes per BAC. The average length of the predicted genes was 1,408 bp. These sequences comprise 37% of the assembly (1 gene per 3.8 kb). After running Blast2GO, 148 sequences were annotated with GO terms (Fig. 3), and 208 sequences exhibited matches against the InterPro collection of protein signature databases.

Metabolic processes were the main biological processes identified among the annotated sequences (83 sequences, Fig. 3A). With respect to molecular functions, most of the sequences (73) exhibited catalytic activity, among which ~ 37% of the catalytic sequences demonstrated hydrolase activity (Fig. 3B).

**Fig 4:**
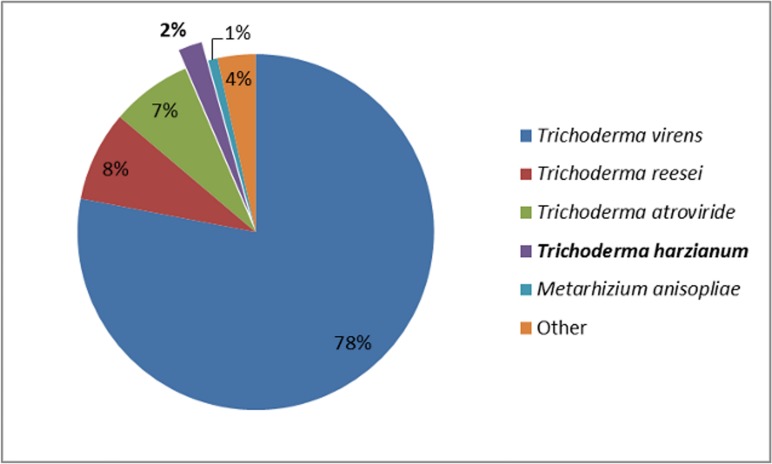
Species distribution. Based on the number of BLAST hits for predicted genes, *T*. *virens* is the species with the most similarity to *T*. *harzianum*.

Regarding the obtained BLAST hits, the species distribution suggested that *Trichoderma virens* is the most similar species to *T*. *harzianum*, showing 181 BLAST hits (78% of the hits), followed by *T*. *reesei* (19 BLAST hits) and *T*. *atroviride* (17 BLAST hits). Although this analysis provides an indication of the similarity between species, there is a discrepancy in the amount of data available for *Trichoderma* species. For instance, *T*. *harzianum* was the *Trichoderma* species presenting the fewest BLAST hits (2%) to *T*. *harzianum* IOC-3844, due to the limited genomic data available for this species (Fig. 4). Thus, BLAST hits should not be used as the single method for determining similarity among *Trichoderma* species.

#### Annotated CAZy genes

**Fig 5:**
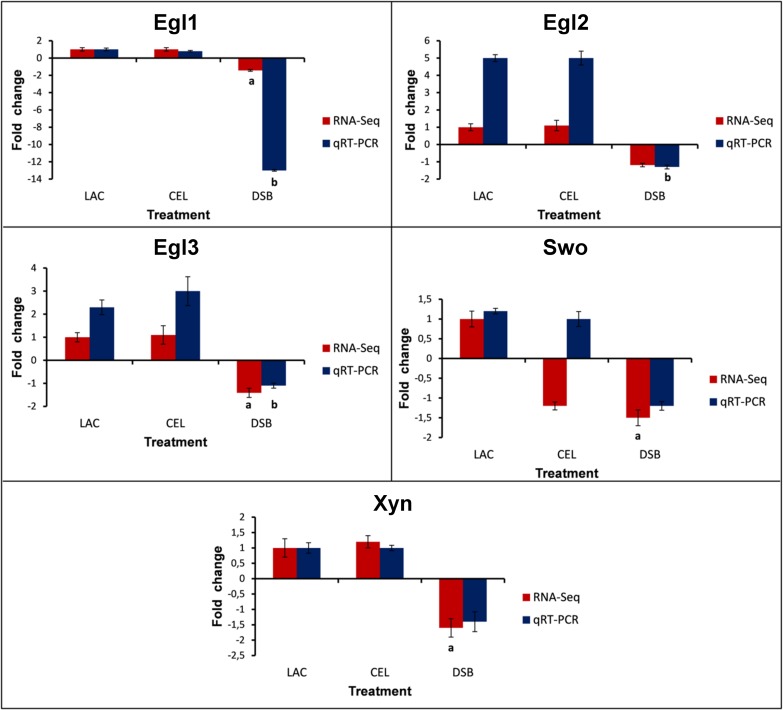
Validation of the RNA-Seq data via qRT-PCR. Expression profiles of the genes related to biomass degradation, as detected through RNA-Seq analysis and validated via qRT-PCR. The squalene epoxidase gene was used as an endogenous control. The differences between the treatments were considered significant at P<0.05 (Student's t-test) and are indicated by (A) comparison with data obtained in the RNA-Seq analysis and (B) comparison with data obtained in the qRT-PCR analysis. The error bars indicate the standard deviation of three biological replicates.

Although we began using 7 CAZy genes for BAC screening and sequencing, we identified 17 CAZy genes in the assembled BAC sequences (Fig. 5; Table 2). The Bgl2 BAC was the only BAC containing a single CAZy gene.

**Table 2:** CAZy genes identified among the assembled BAC sequences.

**BAC**	**CAZy Family**	**Enzyme**	**EC Number**
***egl1***	GT71	α-mannosyltransferase	2.4.1.-
	GH7	endoglucanase I	3.2.1.4
***egl2***	GH3	β-xylosidase	3.2.1.37
	GH5	endoglucanase II	3.2.1.4
	GH79	endo-β-N-glucuronidase/heparanase	3.2.1.-; 3.2.1.166
***egl3***	CE16	acetylesterase	3.1.1.6
	GT90	O-β-glucosyltransferase	2.4.1.-
	GH12	endoglucanase III	3.2.1.4
	GH37	α,α-trehalase	3.2.1.28
	GT17	GnT-III (β-1,4-N-acetylglucosaminyltransferase III)	2.4.1.144
***bgl2***	GH1	b-glucosidase 2	3.2.1.21
***cbh1***	GH92	α-mannosidase	3.2.1.-
	GH7	cellobiohydrolase I	3.2.1.91
	CBM_1	swollenin	-
	GH5	xylanase	3.2.1.45
***xyn2***	GH27	α-galactosidase	3.2.1.22
	GH11	xylanase	3.2.1.8

The Egl3 BAC exhibited the highest concentration of CAZy genes, whereas the Bgl2 BAC only contained 1 sequence belonging to the CAZy group.

CAZymes play a crucial role in biomass degradation, and CAZy genes are not distributed randomly in the *T*. *reesei* genome [30]. Considering a genome size of 40 Mbp (according to the sequenced *T*. *harzianum* CBS 226.95 strain) and 487 CAZy genes, as predicted in a previous transcriptome study [16], we expected to identify 1 CAZy gene per ~82 kb. In the present analysis, we found an average of 1 CAZy sequence per ~18 kb, which represents a 4.5-fold increase in the number of expected CAZy genes. This assay confirms the findings of a previous study in which CAZymes were analyzed in the *T*. *reesei* genome [30]. In this previous study, 41% of the *T*. *reesei* CAZy genes were identified in 25 discrete genome regions, ranging from 14 to 275 kb in length. This density is approximately five-fold higher than would be expected for randomly distributed genes [30].

The glycoside hydrolase (GH) (EC 3.2.x.y) family represents the major group of CAZy genes that were identified in all of the BAC sequences, with 12 sequences belonging to 10 different GH families. GHs hydrolyze glycosidic bonds between two or more carbohydrates or between a carbohydrate and a non-carbohydrate moiety (CAZY), thereby playing an important role in the degradation of cellulose and hemicellulose compounds. Detailed information regarding each of the identified CAZy genes is provided in the following sections.

Three identified CAZy genes belong to the glycosyltransferase (GT) group. GTs (EC 2.4.x.y) are involved in the biosynthesis of disaccharides, oligosaccharides, and polysaccharides [31]. Although few GTs were identified in the BAC sequences compared with GHs, all of the GTs were related to fungal cell wall synthesis. Previous studies have demonstrated the genomic co-localization of GTs involved in cell wall synthesis with GHs related to biomass degradation [30]. However, no report has yet explained the possible functional correlation between these two classes of enzymes. Here, we also report the co-induction of a GT with a cellulase gene, with both belonging to the same BAC, as shown below.

One predicted CAZy gene identified in the Cbh1 BAC was classified as containing a carbohydrate-binding module family 1 (CBM_1) member that was identified as a swollenin. Swollenin is an expansin-like protein that was first identified in *T*. *reesei* and disrupts cellulose fibers [32]. Expansins weaken the non-covalent interactions of plant cell walls, inducing their extension [33]. This role was also confirmed for fungal swollenin. Specifically, activity assays performed on cotton fibers and filter paper detected disruption and weakening of cellulose, without the release of reducing sugars [32]. As the disruption of the cellulose structure boosts the activity of hydrolytic enzymes by enhancing the access of these enzymes to the fibers, swollenin potentially plays a role in assisting hydrolysis by GHs [34].

We also identified a carbohydrate esterase (CE) from family 16, an acetylesterase (EC 3.1.1.6) that catalyzes the hydrolysis of acetyl side groups from glucuronoxylan, thus participating in the biodegradation of xylan [35].

### Real-time quantitative qRT-PCR analysis

Some predicted genes obtained through the analysis of RNA-Seq data were previously selected for validation via qRT-PCR [16]. In this work, we validated the expression of an additional five (*egl1*, *egl2*, *egl3*, *swo* and *xyn2*) selected genes through qRT-PCR. The genes and their respective primers are presented in S2 Table of the Supporting Information.

The results are consistent with those obtained from the RNA-Seq data, given that similar expression profiles were observed in the two analyses. The single exception was the expression profile of *swo* in the CEL treatment. Although *swo* presented opposite expression profiles between the two techniques, its expression profile may be a “borderline” expression profile due to the low fold changes (-1.2 for RNA-Seq and 1.0 for qRT-PCR) observed in both situations. Additionally, the difference in expression levels observed for some genes (especially for *egl1*, which presented a difference of ~ 6 fold) between the two techniques may be explained by the superior sensibility of qRT-PCR in the quantification of gene expression compared with RNA-Seq [36, 37].

Regarding the expression levels determined under the different treatments (LAC, CEL and DSB), using LAC as the control condition, the genes analyzed via qRT-PCR presented a lower expression level in the DSB treatment, which is consistent with the RNA-Seq data. Fig. 5 presents the relative quantification (RQ) results for selected genes under the three conditions (LAC, CEL and DSB).

### CAZy gene expression levels

**Fig 6:**
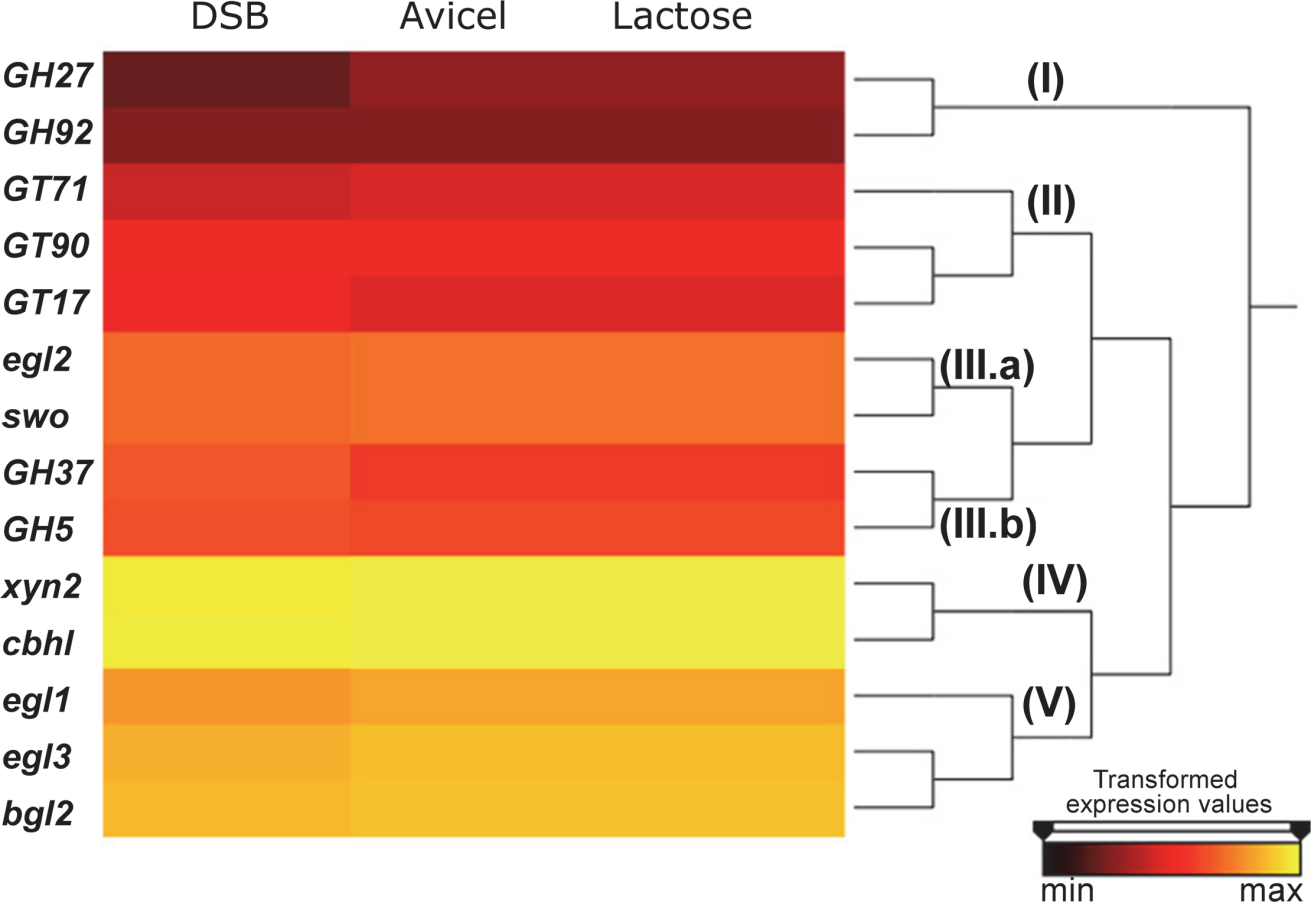
Hierarchical clustering of CAZy genes. CAZy sequences were mapped against transcriptome reads and divided into 5 transcript groups according to expression levels. Group IV contains the most highly expressed sequences, and group I contains the sequences with the lowest expression.

We analyzed the expression levels of 17 CAZy genes using hierarchical clustering (Fig. 6), and the genes clustered into 6 transcript groups.

Group I contained the CAZy genes exhibiting minimal expression among those analyzed in the present study, including the α-galactosidase (GH27) gene from the Xyn2 BAC and the α-1,2-mannosidase (GH92) gene from the Cbh1 BAC. Group II consisted of the 3 glycosyltransferases: α-mannosyltransferase (GT71) from the Egl1 BAC, a candidate β-xylosyltransferase (GT90) from the Egl3 BAC, and GnT-III (β-1,4-N-acetylglucosaminyltransferase III, GT17) from the Egl3 BAC. All of the identified GTs were included in the same group given their similar expression levels under various conditions.

Group III was formed by two sub-groups. Group IIIa was composed of *egl2* and *swo*, whereas group IIIb was formed by a GH37 family protein (α,α-trehalase) from the Egl3 BAC and a candidate xylanase from the Cbh1 BAC.

Group IV included the genes with the highest expression: *xyn2* and *cbh1*, a hemicellulase and a cellobiohydrolase, respectively. Finally, group V contained *egl1*, *egl3* (exhibiting the highest expression among the 3 analyzed endoglucanases), and *bgl2*.

Egl3 and is the only endoglucanase that lacks a CBM (cellulose-binging module), which is responsible for recognizing and binding to the surface of cellulose. In addition, it has been shown that removal of the CBM of cellulases reduces the hydrolytic activity of the catalytic module on crystalline substrates, such as Avicel (microcrystalline cellulose) [38]. A previous study demonstrated that Egl3 adsorbs to both Avicel and phosphoric acid swollen cellulose (PASC), though the observed affinity and hydrolytic properties were weak compared with those of the other cellulases [39]. In *T*. *reesei*, the best-studied endoglucanases include Egl1 and Egl2, which are the most abundant of the endoglucanases produced, with Egl2 playing a major role in the total hydrolysis of cellulose [39–41]. Intriguingly, in *T*. *harzianum* IOC-3844, *egl3* exhibited the highest expression among the endoglucanases analyzed in this study in all treatments (DSB, CEL and LAC).

### Screening of candidate genes related to biomass hydrolysis

Cellulase and hemicellulase production is an energy-consuming process for the fungus; therefore, the genes encoding these enzymes are tightly regulated [42]. Synergistic action of the enzymes is required for the degradation of carbohydrate polymers [43], and thus, the coordination of their expression is highly dependent on inducers and repressors. In addition, the direct or indirect participation of several CAZy genes involved in biomass degradation is not fully understood.

Because genes showing similar expression profiles under different conditions often participate in the same molecular pathway, we analyzed the expression profiles of the genes obtained in the BAC library and compared them with those of the CAZy genes to gain further insight regarding their potential role in biomass hydrolysis. We further considered the vicinity of the genes. The BAC genes were mapped against transcriptome reads to analyze their expression profiles under the three treatments; each BAC was analyzed individually. The genes presenting the same expression profile as the CAZy genes from the same BAC were identified through K-means clustering analysis, and their possible influence on biomass hydrolysis is discussed in the following subtopics. Table 3 presents the clusters formed by the CAZy and non-CAZy genes that exhibited similar expression profiles.

**Table 3:** K-means clustering of the BAC genes.

**Cluster—**CAZyme (Family)	**Co-induced gene**	**Encoded protein/function**	**InterPro**
**A**—Endoglucanase 1 (GH7)	GT71	mannosyltransferase	22751
**B**—Endoglucanase 2 (GH5)	2.7	α-β hydrolase fold	29058
	2.28	major facilitator superfamily	11701
	2.43	oxidoreductase activity	01128
	2.48	carboxylesterase	02018
**C-** β-xylosidase (GH3)	2.2	Zn(2)-C6 transcription factor	01138
	2.11	cysteine synthase	01926
	2.49	ribonuclease activity	16191
**D**—Heparanase (GH79)	2.6	amidohydrolase	17439
**E**—Endoglucanase 3 (GH12)	3.14	metallopeptidase activity	24079
	3.23	serine-type endopeptidase activity	15500
	3.25	amino acid permease	04762
	3.33	Zn(2)-C6 transcription factor	27392
**F**—Trehalase (GH37)	3.19	protein kinase binding	15431
	3.27	related to conidiation	-
**G**—GnT-III (GT17)	3.20	N-acetyltransferase activity	00182
	3.37	GTP binding	06689
	3.45	protein kinase binding	13922
**H-**Xylosyltransferase (GT90)	3.2	b-ketoacyl synthase	20801
	3.5	GTP binding	06689
	3.42	DNA-dependent RNA polymerase	07120
**I-** β-glucosidase 2 (GH1)	b.7	Zn(2)-C6 transcription factor	01138
	b.8	5-aminolevulinate synthase	01917
	b.30	cation transporter	03445
**J—**Cellobiohydrolase I (GH7)	*swo*	swollenin (expansin-like)	07117
**K—**Xylanase (GH5)	c.7	NAD(P)-binding domain	16040
	c.12	ribonuclease E inhibitor	05493
	c.18	major facilitator superfamily	05828
	c.31	α-β hydrolase fold	29058
	c.32	oxidoreductase activity	03042
	c.42	cytoskeleton-associated	-
**L—**Mannosidase (GH92)	c.11	sugar/inositol transporter	03663
	c.13	transcription factor domain	07219
	c.17	oxidoreductase activity	03042
**M**—Xylanase (GH11)	x.13	pth11-type coupled receptor	-
**N-** α-galactosidase (GH27)	x.16	cyclopropane fatty acid synthase	20803

The predicted genes with expression profiles similar to CAZy are listed. Each BAC was divided into 10 clusters, and clusters containing at least one CAZy are summarized.

#### Endoglucanase I BAC

**Fig 7:**
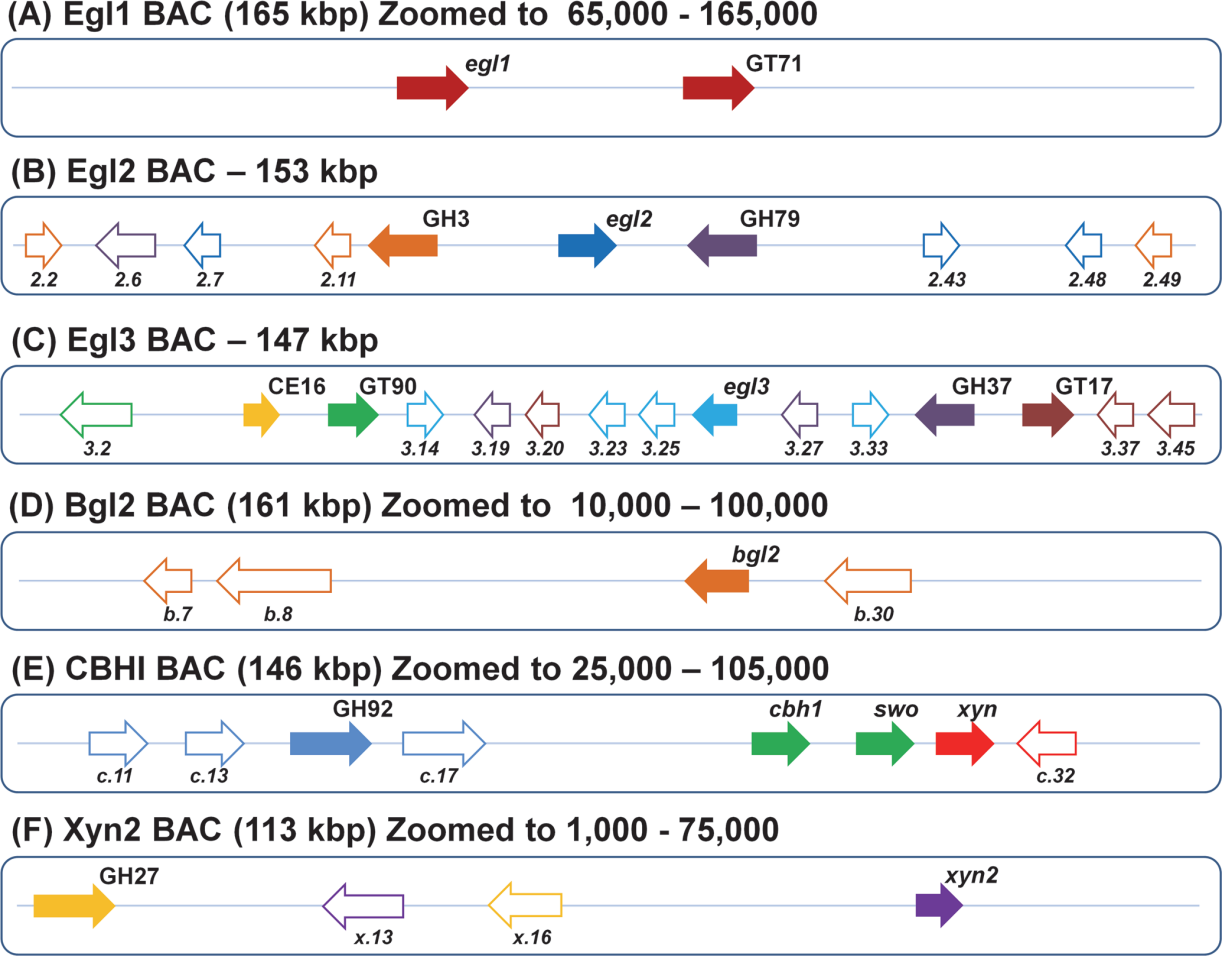
Schematic model of CAZy and co-regulated genes. CAZy genes are indicated with color-filled arrows. Co-regulated non-CAZy genes are indicated by white arrows with contours of the same color as those of CAZy genes with similar profiles. Similar colors indicate a similar expression profile. (A) The region of the Egl1 BAC containing Egl1 and a gene encoding a GH71 family protein, which exhibit similar expression profiles. (B) The region of the Egl2 BAC containing a GH3 family protein, an Egl2 gene, and a GH79 protein. (C) The region of the Egl3 BAC containing 5 CAZy genes: CE16, GT90, Egl3, CH37, and GT17. (D) The region of the Bgl2 BAC that contains only one CAZy, the Bgl2 gene. (E) The Cbh1 BAC, demonstrating that Cbh1 and swollenin are co-induced. Two other CAZy genes (GH92 and GCase) are also present. (F) The region of the Xyn2 BAC containing a GH27 protein and *Xy2*.

The Egl1 BAC (165 kbp) showed 2 CAZymes: a GT from family 71 and a GH from family 7 (a predicted α-mannosyltransferase and Egl1, respectively) (Fig. 7A). Intriguingly, the two CAZymes formed a cluster, suggesting co-regulation.

Endoglucanase I is a cellulase that catalyzes the endohydrolysis of (1→4)-β-D-glucosidic linkages; it exhibits activity against cellulose, 1,3–1,4-β-D-glucan, xyloglucan, xylan, and mannan [44]. The distance between the GH7 gene and the GT71 gene was 23 kb, indicating close proximity. GT71, an α-mannosyltransferase, contains a transmembrane region and is involved in the incorporation of glycoproteins into the cell wall of *S*. *cerevisiae* [45]. In addition, it is implicated in late Golgi modifications [46]. As previously reported, GTs related to cell wall synthesis are occasionally located in genomic regions containing GHs involved in biomass degradation. A previous study demonstrated that a putative α-1,2-mannosyltransferase is required for cell wall stability and virulence in *Aspergillus fumigatus*. During growth, the fungal cell wall must be reorganized, and mannosyltransferases play a crucial role in this process because they are involved in glycosylation [47]. Several cellulases and hemicellulases are secreted via the action of transport-related proteins, whereas others remain bound to the cell wall, at least for a short period of time [48]. Thus, an optimal rearrangement of the cell wall during fungal growth is essential for biomass degradation because defects in protein glycosylation may result in misfolding, instability, and reduced enzymatic activity [47]. These requirements may explain the presence of GTs such as α-mannosyltransferase near certain cellulase-coding genes.

#### Endoglucanase II BAC

The BAC corresponding to Egl2 (153 kb) exhibited 3 glycosyl hydrolases: a GH5 family Egl2, a candidate β-xylosidase from the GH3 family, and a GH79 family protein with endo-β-N-glucuronidase or heparanase activity (Fig. 7B).

Cluster B (Table 3), containing *egl2*, included a gene encoding a candidate D-galactonate transporter that belongs to the major facilitator superfamily (MFS). Transporter proteins are relevant for the utilization of carbon sources, and the co-induction of transport genes with CAZy genes located in the same genomic region is common [30].

Cluster C contained a GH3 protein (Table 3). This cluster was constructed using data from two of the three treatments (DSB and lactose) because no matches to the reads obtained under cellulose induction were identified. Given that the gene product is a xylosidase acting on xylans and xylobiose, no mapped reads were noted when crystalline cellulose was used as the induction substrate. Gene 2.2 from cluster B (Fig. 7B) is a protein with a Zn(2)-C6 fungal-type DNA-binding domain (IPR001138). A previous study [49] analyzed candidate cellulase/hemicellulase regulator genes from *T*. *reesei* under various conditions. This previous study identified numerous genes encoding putative fungal C6 zinc finger-type transcription factors, including several from IPR001138, that were co-regulated with cellulase and/or hemicellulase genes.

#### Endoglucanase III BAC

The BAC that was sequenced from the Egl3 gene contained a 147-kbp region with 5 CAZy genes, which was the most CAZy-rich region among the six BACs studied in the present work. The following 5 CAZy genes were noted in this region: 2 GT family proteins (GT17 and GT90), 2 GH family proteins (GH12 (i.e., *egl3*) and GH37), and 1 CE family protein (CE16). CE16, an acetylesterase, did not form a cluster with other genes from the Egl3 BAC, exhibiting a unique expression profile (Fig. 7C).

Here, we emphasize the relevance of transporter genes, as they clustered with several CAZy genes in the present study. In cluster E (Table 3), an amino acid permease gene (3.25) was consecutive to *egl3*; these genes are separated by a distance of < 200 bp (Fig. 7C). Additionally, another Zn(2)-C6 transcription factor was identified that exhibited an expression profile similar to *egl3*. GH 37, an α,α-trehalase, formed a cluster (F, Table 3) with a hypothetical protein related to conidiation. In fact, the development of the fungal mycelium and conidiation are associated with increased trehalase activity [50]. GT 17, a GnT-III (cluster G, Table 3), formed a cluster with an n-acetyltransferase gene. Six genes belonging to this class are co-regulated with cellulase and/or hemicellulose genes in *T*. *reesei*, in which one of these genes was overexpressed in a mutant strain, resulting in an increase in cellulase/hemicellulase activity [49].

#### β-Glucosidase BAC

The BAC containing the β-glucosidase gene *bgl2* was the only BAC showing a single CAZyme, Bgl2 (Fig. 7D).

Based on observing the same expression profile as *bgl2* (cluster I, Table 3), we were able to identify a transcription factor containing a Zn(2)-C6 fungal-type binding domain and a transporter gene (b.30; Fig. 7D) located < 10 kbp from *bgl2*.

#### Cellobiohydrolase/Swollenin BAC

The BAC containing *cbh1* (GH7) presented 4 CAZy genes: 3 GHs and 1 protein containing a CBM (swollenin). This BAC included one of the most intriguing CAZy-enriched regions, with 3 consecutive CAZy genes that are similar to the set of CAZy genes identified in *T*. *reesei*, in terms of both sequence identity and their position within the genome. This group is formed by *cbh1*, *swo*, and a xylanase gene (Fig. 7E).

Cbh1 is one of the most important enzyme components for cellulose depolymerization in fungi. The activity of Cbh1 is essential for the hydrolysis of microcrystalline cellulose and represents 60% of the total cellulase protein produced by *T*. *reesei* by mass [51]. However, Cbh1-mediated cellulose polymerization occurs at a slow rate [52]. The action of Cbh1 is enhanced by synergistic activity with other cellulases, as shown in previous studies [53, 54]. Although *cbh1* and *swo* are in close proximity (separated by a distance of only 8.5 kbp) and are co-induced, as shown in cluster J (Table 3), the synergistic activity between Cbh1 and Swo is poorly understood.

The Cbh1 BAC also contained a putative xylanase that is situated in sequence with *cbh1* and *swo* (Fig. 7E). Although it is consecutive to the other two CAZy genes (which show similar expression profiles), our data indicate a different expression profile for the xylanase, which was separate from *cbh1* and *swo* in the clustering analysis. The xylanase gene belonged to a cluster (K; Table 3) containing 7 total genes, wherein the most similar expression profile was that of gene c.32, a hypothetical protein with oxidoreductase activity.

GH92, an α-1,2-mannosidase, was included in cluster L (Table 3). α-1,2-mannosidase participates in the postsecretory glycosylation of Cbh1 from *T*. *reesei* under various growth conditions [55]. Gene c.11 encodes an integral membrane protein from the sugar/inositol transporter family and is a transporter gene situated near (6 kbp) a CAZy gene. Gene c.13 encodes a transcription factor protein situated 2.6 kbp from GH92; this gene contains a Gal4-like Zn2Cys6 binuclear cluster domain, which is involved in DNA binding.

#### Xylanase BAC

The Xyn2 BAC contained 2 CAZy genes: a GH11 family *xyn2* and a GH27 family α-galactosidase (Fig. 7F).

Xylanase was the biomass degradation-related enzyme that showed the highest expression levels in *T*. *harzianum* IOC-3844 under the 3 analyzed conditions. The xylanase gene clustered with a gene encoding a transmembrane pth11-type coupled receptor protein (M, Table 3). This class of receptors was first identified in *M*. *grisea* [56] and is regulated by LAE1, a global regulator from *T*. *reesei* that is also responsible for regulating the expression of cellulases and polysaccharide hydrolases [57]. A gene encoding a G-protein-coupled receptor was identified in *T*. *atroviride*. Silencing of this gene significantly decreased the growth of β-linked galactooligosaccharides [58]. This feature might be implicated in the lactose induction of *xyr*, the main transactivator of a wide range of cell wall-degrading enzymes, but further studies are required to investigate its function.

### K-means clustering of the CAZy genes

We also verified the expression profile via K-means clustering by exclusively considering the CAZy genes that were identified in all of the BACs. This analysis aimed to demonstrate the co-induction of CAZy genes on the biomass substrate and did not take into account the genomic region in which these genes are inserted. The 17 genes did not form a cluster because some presented a unique expression profile. Here, we discuss 3 different clusters of genes exhibiting similar expression profiles (Table 4).

**Table 4:** K-means clustering of CAZy genes.

**Cluster**	**CAZy (family)**	**CAZyme**
**1**	GH5	xylanase
	GT17	GnT-III
**2**	GH11	xylanase
	GH7	endoglucanase 1
	GH12	endoglucanase 3
	GT71	α-mannosyltransferase
**3**	GH1	β-glucosidase 2
	GH5	endoglucanase 2
	GH7	cellobiohydrolase 1
	CBM_1	swollenin

Ten CAZy genes were divided into 3 clusters to delineate the expression profile, suggesting possible synergism among their encoded proteins.

Cluster 1 (Table 4) was formed by a xylanase (GH5) and a predicted GnT-III (GT17). The second cluster (cluster 2, Table 4) was formed by the Xyn2 gene, Egl1, Egl3, and the predicted α-mannosyltransferase (GT71). These genes were the most highly expressed endoglucanases among the analyzed genes and exhibited an expression profile similar to that of Xyn2, the most highly expressed CAZyme in *T*. *harzianum* IOC-3844 [16].

Finally, the third cluster (3, Table 4) contained the Bgl2, Egl2, Cbh1, and Swo genes. Cbh1 is the major cellulase produced by *T*. *harzianum* and is located near the swollenin gene. A previous study reported a method for the quantitative determination of non-hydrolytic disruptive activity on crystalline cellulose and verified that Egl2, which does not exhibit activity against crystalline cellulose, released reducing sugars in reaction mixtures containing Avicel when swollenin was present [59]. Another study analyzed the synergism between swollenin and other cellulases/hemicellulases. This previous analysis verified an increase in xylose release when swollenin was incubated with Egl2 or with *Xyn10A* and *Xyn11A* (especially with the endoxylanases) using steam-pretreated corn stover as a substrate. However, no synergism with Cbh1 was noted [34].

## Conclusions

This study provides the first BAC-based structural genomic information on the important cellulolytic fungus *T*. *harzianum*. The BAC library provided 12-fold coverage of the *T*. *harzianum* genome and permitted the rapid selection of genes and genomic regions associated with biomass conversion. We identified regions with a high concentration of CAZy genes in this fungus that were previously unknown. An analysis based on transcriptome data, together with the information provided by the BAC library, permitted the screening of candidate genes that may be related to the major cellulases and hemicellulases in *T*. *harzianum*. Transporter genes and transcription factors could play important roles in the expression of CAZymes, as these genes are frequently located in co-regulated genomic regions, flanking CAZy genes. We also analyzed the expression profiles of major cellulase and hemicellulase genes in this strain, revealing a potential synergistic relationship among these genes under the three different analyzed conditions. To our knowledge, this is the first study focusing on the genomic context of biomass degradation genes from *T*. *harzianum*, which is a promising strain for use in biotechnological processes.

## Supporting Information

S1 Table Primers used for the screening of BAC clones. (DOCX)

S2 Table Primers used for the qRT-PCR analysis. (DOCX)
