# Supplementary material for: Analysis of Genomic Regions of Trichoderma harzianum IOC-3844 Related to Biomass Degradation
Source: PLoS One. 2015 Apr 2;10(4):e0122122. doi: 10.1371/journal.pone.0122122 (PMC4383378; doi:10.1371/journal.pone.0122122)
Supplement: S1 Table — (DOCX) [file pone.0122122.s001.docx]

**S1 Table. Primers used for the screening of BAC clones**

| **Gene** | **5' primer** | **3' primer** |
| --- | --- | --- |
| ***egl*1** | CAGTGGCCGTGCAAGA | AACCAGTACATGCCCAGC |
| ***egl*2** | GACTGGACTCAATGGTTCG | CTACCTGTTGCCACTTG |
| ***egl*3** | GTAAACTCACTCAACTCGGC | TCGTAGATAGTTGAAGAAGTT |
| ***cbh*1** | CACGATACCAAGAGCACC | CTATCGGTCATTGGAGTAAGT |
| ***bgl*2** | CCAGAGTTTACGCCCGAG | GCTGCTGTCCATTCTCGTAA |
| ***xyn*2** | ATGGTTGCCTTTACTTCCC | CTGGTTGACACGCTGAGTT |
| ***swo*** | CAATGTGGAGGTAATGGATG | CGAAGAGTAGTGCCCGTAG |
