# Supplementary material for: Analysis of Genomic Regions of Trichoderma harzianum IOC-3844 Related to Biomass Degradation
Source: PLoS One. 2015 Apr 2;10(4):e0122122. doi: 10.1371/journal.pone.0122122 (PMC4383378; doi:10.1371/journal.pone.0122122)
Supplement: S2 Table — (DOCX) [file pone.0122122.s002.docx]

**S2 Table. Primers used for the qRT-PCR analysis**

| **Gene** | **5' primer** | **3' primer** |
| --- | --- | --- |
| ***egl1*** | ACTACCGCTGGATGCACGA | AGACACCAGAGGCAGCGTAGT |
| ***egl2*** | GCCACTACCATGTCTACCTCAAC | TCAGTCCCACAGCCAAAATC |
| ***egl3*** | TCCTTCAGTCAGCAACAATCTCT | GACATTGGGATAGGACTTGACG |
| ***xyn2*** | AATCTACGGTTGGTCTCGCA | CTGCCGTCAGATGTAACCTCA |
| ***swo*** | TGTGTGGAGGTAATGGATGGAC | CCTGAGCTGGATGAAGTTGATG |
| ***erg1*** | GTTGTATGAGACCAGATCGGC | TCACAATGTGTCAATCGAGCA |
